# Frozen-thawed embryo transfer in modified natural cycles: a retrospective analysis of pregnancy outcomes in ovulatory women with vs. without spontaneous luteinizing hormone surge

**DOI:** 10.1186/s12884-022-05161-5

**Published:** 2022-11-04

**Authors:** Hongjuan Ye, Liya Shi, Xinxin Quan, Xue Xue, Ying Qian, Hui Tian, Songguo Xue, Lihua Sun

**Affiliations:** 1 School of Life Sciences and Technology, Tongji University, Shanghai, China; 2 Department of Reproductive Medicine Center, Shanghai East Hospital, Tongji University School of Medicine, Shanghai, China

**Keywords:** Modified natural cycle, Spontaneous LH surge, Frozen-thawed embryo transfer, Pregnancy outcome

## Abstract

**Background:**

Timing of frozen embryo transfer (FET) in natural endometrial preparation cycles is often based on luteinizing hormone (LH) surge. However, some patients do not show spontaneous LH surge despite follicular maturation. The objective of this study was to evaluate the impact of spontaneous LH surge on pregnancy outcomes in modified natural cycles (mNC).

**Methods:**

This retrospective analysis included 1897 FET cycles with modified natural endometrial preparation in normo-ovulatory women between January 1, 2015, to December 31, 2019, at our center: 920 cycles with spontaneous LH surge (≥ 20 IU/L) and 977 without. For cleavage embryos, FET was conducted 4 and 5 days after hCG injection in women with and without LH surge, respectively. For blastocysts, FET was conducted 6 and 7 days after hCG injection in women with and without LH surge, respectively. Multivariate regression was conducted to examine the factors associated with live birth.

**Results:**

Live birth rate was 43.7% in patients with spontaneous LH surge vs. 43.8% in women without LH surge (*P* = 0.961). The two groups also had similar implantation rate (36.2% vs. 36.7%, *P* = 0.772), biochemical pregnancy rate (54.8% vs. 55.4%, *P* = 0.796) and clinical pregnancy rate (50.9% vs. 51.7%, *P* = 0.721). In multivariate regression, live birth was not associated with LH surge (aOR, 0.947, 95% CI, 0.769, 1.166).

**Conclusion:**

Pregnancy outcomes were similar in mNC-FET in cycles with vs. without spontaneous LH surge if FET timing is adjusted.

## Background

With the advances in cryopreservation technology, excessive embryos could be more safely frozen and preserved for later use [1]. With the freeze-all strategy, the best embryos can be transferred into a more receptive endometrium rather than immediately to avoid the potential endometrial damage by the controlled ovarian stimulation (COS) [2]. In a large, multicenter, randomized trial involving 1650 ovulatory infertile women, frozen blastocyst transfer resulted in a higher live birth rate than fresh blastocyst transfer [3].

Endometrial preparation prior to embryo transfer is critical for achieving live birth [4]. Previous studies have evaluated different endometrial preparation protocols, including natural cycle, ovulation stimulation (OS) cycle with drugs (e.g., letrozole or gonadotrophin), or artificial cycle [5, 6], but did not provide conclusive evidence to support the advantage of OS and artificial cycle over the natural cycle with respect to pregnancy outcomes [7–9]. For women with regular and ovulatory cycles, the natural cycle is preferred by the physicians and patients, but requires frequent ultrasound examination and blood hormone test for monitoring spontaneous luteinizing hormone (LH) surge [10], as well as an estimated 6% cycle cancellation rate [11]. Modified natural cycle (mNC) could avoid the cancel and reduce the requirement for visit, in which human chorionic gonadotropin (hCG) was used to simulate LH surge in the frozen embryo transfer (FET) cycle when dominant follicle appeared.

A recent retrospective cohort study that included 1937 women failed to show significant difference in the live birth rate between ovulation with spontaneous LH surge or hCG trigger [12]. But other studies comparing hCG triggering ovulation with spontaneous LH surge ovulation yielded controversial results [13–15], possibly due to complex interaction between spontaneous LH surge and hCG as well less-than-optimal synchronization of embryo development and endometrium [16]. In these studies, although the final ovulation outcome was the same, there were two independent variables: spontaneous LH surge and hCG trigger, and whether hCG itself had an effect on pregnancy outcome, improved or deteriorate, was no consensus yet [12, 17, 18].

To eliminate the potential bias of hCG itself on pregnancy outcome in the FET cycle, we conducted a retrospective analysis to examine pregnancy outcomes with or without LH surge in women receiving FET with the mNC.

## Materials and methods

### Study design

This retrospective study was approved by the Institutional Review Board of Shanghai Ninth People’s Hospital (Number: 2013–46) and was conducted in compliance with the Declaration of Helsinki.

**Fig. 1:**
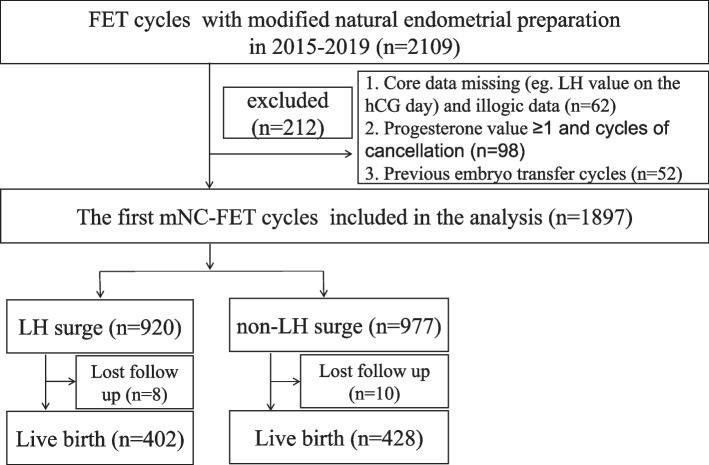
Selection process of the cases included in the final analysis

This analysis included regular menstruating (25–35 days in menstrual duration) women who underwent their first mNC-FET between January 1, 2015, to December 31, 2019, at the Department of Assisted Reproduction, Shanghai Ninth People's Hospital affiliated to Shanghai Jiaotong University School of Medicine. The patient selection process is presented in Fig. 1. Exclusion criteria included: 1) previous history of embryo transfer cycles; 2) missed serum LH value and progesterone level ≥ 1 ng/ml on the hCG day, and illogical data; 3) canceled cycles due to spontaneous ovulation without signs of premature luteinization and no embryos transferred after warming failure.

### COS and embryo quality assessment

COS protocol was progestin-primed ovarian stimulation protocol and antagonist protocol for the regular menstruation cycle in our centre [19, 20]. The choice of conventional in vitro fertilization (IVF) vs. intracytoplasmic sperm injection (ICSI) was performed based on semen parameters and previous fertilization history, as previously described [19, 20]. Embryos were cultured in Continuous Single Culture (Irvine Scientific, USA) throughout the entire development stage. Culture conditions remained identical during the study period. Embryos were vitrified and thawed as previously described [21].

Cleavage embryo quality was assessed based on the Cummins’ criteria [22] into four grades. Grade I and II were considered good-quality embryos. Blastocyst quality was assessed using the Gardner and Schoolcraft system [23]. All embryos were vitrified and frozen for subsequent transfer. Embryos with good quality were transferred first; embryos with poor quality were considered if embryos with good quality were not (or no longer) available. One or two embryos were transferred each cycle.

### MNC-FET and luteal phase support

Baseline follicle development (using transvaginal ultrasound) and serum hormones were examined on day 3 in the menstrual cycle to eliminate the ovarian cyst. All the blood tests were performed using the Chemiluminescent method (Abbott Biologicals B.V., the Netherlands), and the follicular monitoring was conducted by the experienced physicians. In order to reduce the hospital visits, a urinary LH test daily at home was required [24]. If the dominant follicle was ≥ 10 mm on day 10, patients were asked to conduct daily urinary LH test at home starting from day 11 and come back on the same day of positive urine test; upon negative testing, patients were asked to come back for examination at least every other day. When the leading follicle diameter reached 15 mm and the endometrial thickness was at least 7 mm, the timing of embryo transfer and luteal phase support were performed depending on the serum LH level as follows:

In subjects with LH surge, hCG (6000 IU, intramuscularly) was given on the same day. Luteal phase support using exogenous progesterone (40-mg dydrogesterone plus 0.4-g vaginal micronized progesterone per day) started 2 days after hCG administration. Embryo thaw and the transfer were conducted 4 days after LH surge for cleavage embryos and 6 days after LH surge for blastocysts.

In subjects without LH surge, hCG (6000 IU) was given on the same day of LH monitoring. Luteal phase support started 3 days after hCG administration. Such timing was based on a 1-day delay of ovulation in patients without LH surge vs. with LH surge, as reported previously [25]. Cleavage embryos were thawed and transferred 5 days after hCG administration; the thaw and transfer of blastocysts were scheduled 7 days later.

**Fig. 2:**
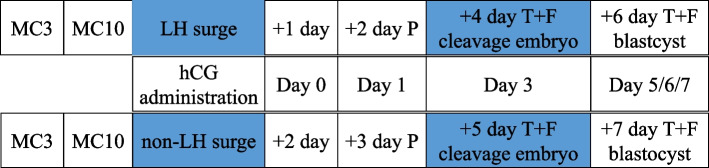
Schematic summary of the FET cycle. Abbreviations: MC3/10, menstrual cycle day 3/10; P, additional progesterone administration; T + F, thawing and frozen embryo transfer; Day 0, theoretical day of ovulation

The entire scheme of FET process is schematically presented in Fig. 2. Blood hormones and ultrasound were not examined after hCG day until on the FET day. Cycles were not canceled if endometrium thickness decreased to lower than 7 mm on the FET day. If pregnancy was achieved, luteal phase support continued until 10 weeks of gestation.

### Outcome measures

The primary outcome measure was live birth rate. A live birth was defined as the delivery of a living newborn after the 24^th^ gestational week. Secondary outcome measures included implantation rate, biochemical pregnancy, clinical pregnancy, pregnancy loss and ectopic pregnancy. Implantation rate was defined as the number of gestational sacs visualized on ultrasound examination divided by the number of embryos transferred. Biochemical pregnancy was defined as serum β-hCG ≥ 25 IU/L at 2 weeks after embryo transfer and there was no gestation sac developed. Clinical pregnancy was defined as at least one gestational sac on ultrasound scanning 4 weeks after embryo transfer. Pregnancy loss including the first-trimester and late pregnancy before the 24^th^ gestational week was defined as a loss of entire gestation.

### Statistical analysis

Distribution of continuous variables was assessed by the Shapiro–Wilk test. Continuous variables were presented as mean ± standard deviation (SD), and compared between subjects with vs. without LH surge using Student's t-test and Mann–Whitney U test. Categorical variables were presented as number and percentage n (%), and analyzed using the chi-squared test. Multivariate logistic regression model was used to identify factors associated with live birth; the result are shown as adjusted odds ratios (aORs) and 95% confidence intervals (CIs). All statistical analyses were performed using the Statistical Package for Social Sciences (SPSS) version 22.0. Statistical significance was set at *P* < 0.05.

## Results

### Patient characteristics

A total of 2109 normal-ovulatory women who underwent FET cycle with mNC were screened during this study period. Of these, 212 were excluded from the final analysis for the following reasons: missing LH value on the hCG day and illogical data (*n* = 62); previous history of embryo transfer (including fresh and/or frozen) (*n* = 52); progesterone value ≥ 1 ng/ml and cycles of cancellation (*n* = 98). 1897 patients who underwent their first mNC-FET were included in the final analysis. The mean age at oocyte retrieval was 33.32 ± 4.62 years. The body mass index (BMI) was 21.42 ± 5.52 kg/m^2^ and endometrium thickness was 10.75 ± 2.08 mm. Of the 1897 cycles, 1387 (73.12%) cycles involved two embryos transfers, and 1585 (83.55%) cycles involved cleavage embryos. None of the patients were smokers in this study.

Characteristics of the patients and cycles are shown in Table 1. Patients with LH surge were similar to those without LH surge in maternal age, BMI, infertility type/cause/duration, antral follicle counts, basal hormonal levels, hormonal levels on the FET day, endometrium thickness, type of fertilization (IVF vs. ICSI), cryopreservation time, the number of embryo transfer, embryo stage and quality. On the hCG day, the LH surge group had higher serum FSH, LH, and progesterone but lower estradiol level (257.99 ± 91.39 vs. 270.18 ± 94.02 pg/ml). Serum estradiol and progesterone level on the FET day did not differ between the 2 groups.

**Table 1:** Characteristics of the patients and cycles

	**Overall sample (*n* = 1897)**	**LH surge (*n* = 920)**	**Without LH surge (*n* = 977)**	***P***
Age (years) mean ± SD	33.32 ± 4.62	33.07 ± 4.56	33.55 ± 4.67	0.258
≥ 37, n (%)	446 (23.51%)	198 (21.5%)	248 (25.4%)	0.584
BMI (kg/m2) mean ± SD	21.42 ± 5.52	21.38 ± 2.76	21.55 ± 7.1	0.226
18, n (%)	156 (8.22%)	75 (8.15%)	81 (8.29%)	
18 ≤ ~ < 24, n (%)	1450 (76.43%)	705 (76.63%)	745 (76.26%)	
24 ≤ ~ < 28, n (%)	242 (12.75%)	118 (12.83%)	124 (12.69%)	
≥ 28, n (%)	49 (2.6%)	22 (2.39%)	27 (2.76%)	
Infertility type, n (%)				0.781
Primary	1000 (52.71%)	488 (53.1%)	512 (52.4%)	
Secondary	897 (47.29%)	432 (46.9%)	465 (47.6%)	
Infertility cause, n (%)				0.663
Tubal	1252 (66%)	618 (67.2%)	634 (64.9%)	
Decreased ovarian reserve	74 (3.9%)	36 (3.9%)	38 (3.9%)	
Endometriosis	231 (12.18%)	111 (12.1%)	120 (12.3%)	
Male factors	279 (14.71%)	118 (12.8%)	161 (16.5%)	
Others	61 (3.21%)	37 (4%)	24 (2.4%)	
Infertility duration (years), median (range)	2 (0–20)	2 (0–20)	3 (0–19)	0.711
Antral follicle counts, mean ± SD	6.19 ± 5.8	6.24 ± 5.85	6.13 ± 5.76	0.221
Basal hormone level, mean mean ± SD				
FSH (IU/L)	5.15 ± 1.62	4.93 ± 1.20	5.73 ± 1.26	0.462
LH (IU/L)	5.24 ± 2.36	5.78 ± 1.99	4.21 ± 2.23	0.567
E2 (pg/ml)	36.98 ± 14.53	36.31 ± 11.40	38.63 ± 17.57	0.564
Progesterone (ng/ml)	0.28 ± 0.14	0.25 ± 0.11	0.32 ± 0.15	0.187
Hormone level, hCG day, mean ± SD				
FSH (IU/L)	7.48 ± 3.96	10.06 ± 3.92	5.1 ± 1.94	< 0.001
LH (IU/L)	23.6 ± 14.44	35.08 ± 12.6	12.8 ± 3.85	< 0.001
E2 (pg/ml)	264.26 ± 92.93	257.99 ± 91.39	270.18 ± 94.02	0.909
Progesterone (ng/ml)	0.45 ± 0.23	0.59 ± 0.21	0.32 ± 0.16	< 0.001
Hormone level, FET day, mean ± SD				
E2 (pg/ml)	115.1 ± 52.23	110.7 ± 47.73	119.07 ± 55.73	0.186
Progesterone (ng/ml)	18.33 ± 7.2	18.02 ± 6.93	18.61 ± 7.43	0.127
Endometrium thickness (mm) mean ± SD	10.75 ± 2.08	10.8 ± 2.1	10.7 ± 2.1	0.283
7, n (%)	114 (6%)	56 (6.09%)	58 (5.94%)	
Fertilization type, n (%)				0.613
IVF	1275 (67.21%)	621 (67.5%)	654 (67%)	
ICSI	622 (32.79%)	299 (32.5%)	323 (33%)	
Cryopreservation time (day), mean ± SD	72.73 ± 2.87	73.26 ± 2.59	72.47 ± 3.11	0.663
Number of embryo transferred, n (%)				0.282
1	510 (26.88%)	236 (25.65%)	274 (28.05%)	
2	1387 (73.12%)	684 (74.35%)	703 (71.95%)	
Embryo stage, n (%)				0.336
Cleavage	1585 (83.55%)	758 (82.39%)	827 (84.65%)	
Blastocyst	312 (16.45%)	162 (17.61%)	150 (15.35%)	
Embryo quality, good (%)	96.3%	96.5%	96.2%	0.236

### Pregnancy Outcomes

Table 2 showed the maternal and neonatal outcomes. Among the 1897 cycles (1897 women), spontaneous LH surge was detected in 920 (48.5%) cycles. A total of 1624 embryos were thawed and 1604 embryos survived in the LH surge group. 1705 embryos were thawed and 1680 embryos survived in the non-LH surge group. 8 patients were lost to follow-up in the LH surge group and 6 patients were in the non-LH surge group. Live birth rate was 43.7% in the LH surge group vs. 43.8% in the non-LH surge group (*P* = 0.961). Other pregnancy outcomes were also comparable between the two groups: including implantation rate (36.2% vs. 36.7%; *P* = 0.772). biochemical pregnancy rate (54.8% vs. 55.4%; *P* = 0.796) and clinical pregnancy rate (50.9% vs. 51.7%, *P* = 0.721). The two groups were also similar in neonatal outcomes.

**Table 2:** Pregnancy and newborn outcomes in two groups in FET cycle

	**LH surge** **(*n* = 920)**	**Without LH surge (*n* = 977)**	***P***
No. of thawed embryos (n)	1624	1705	
No. of viable embryos after thawed (n)	1604	1680	
Implantation, % (n)	36.2% (580)	36.7% (618)	0.772
Biochemical pregnancy, % (n)	54.8% (504)	55.4% (541)	0.796
Clinical pregnancy, % (n)	50.9% (468)	51.7% (505)	0.721
Multiple pregnancy, % (n)	0	0.4% (2)	0.5
Miscarriage, % (n)	11.5% (54)	11.5% (58)	0.979
Heterotopic pregnancy, % (n)	14.9% (7)	21.8% (11)	0.482
Live birth, % (n)	43.7% (402)	43.8% (428)	0.961
Gestation age (weeks)	38.06 ± 1.83	38.14 ± 1.61	0.506
Modes of delivery, n			0.691
Vaginal	119	124	
Cesarean	283	304	
Newborn, n			0.804
Single birth	304	321	
Twin birth	98	107	
Birth weight, mean ± SD			
Singletons	3366.32 ± 450.92	3334.33 ± 453.45	0.794
< 2500 g, n	8	11	
≥ 4000 g, n	21	25	
Twins	2609.04 ± 425.60	2616.61 ± 362.16	0.165
< 2500 g, n	64	74	
< 1500 g, n	2	0	

The following 11 independent variables were entered into the multivariate regression analysis of live birth: age, BMI (underweight, normal, overweight or obese), infertility duration (< 1 vs. ≥ 1 year), infertility type (primary vs. secondary), infertility cause, endometrial thickness, number, stage and quality of transferred embryos, cryopreservation time, and LH surge (yes vs. no) (Table 3). The analysis showed association between live birth with younger maternal age (aOR, 1.088 per every 1-year decrease; 95%CI, 1.059, 1.117; *P* < 0.001), BMI (aOR vs. normal weight, 0.611 for overweight; 95%CI, 0.364, 1.03, *P* = 0.062; 0.504 for obesity; 95%CI, 0.31, 0.505, *P* = 0.006;), endometrial thickness (aOR, 1.054 for every 1-mm increase; 95%CI, 1.001, 1.109; *P* = 0.045), the number of embryos transferred (aOR, 2.454 for 2; 95%CI, 1.846, 3.261; *P* < 0.001), and embryo quality (aOR, 1.035, 95% CI, 1.012, 1.081, *P* = 0.038). Live birth was not associated LH surge (aOR, 0.947, 95% CI, 0.769, 1.166, *P* = 0.607).

**Table 3:** Multivariate regression analysis of the factors associated with live birth

	aOR (95%CI)	*P*
Age (years)	0.919 (0.895, 0.944) per every 1-year increase	< 0.0001
BMI (kg/m2)		
< 18	1.448 (0.837, 2.503)	0.185
18 ≤ ~ < 24	reference	
24 ≤ ~ < 28	0.611 (0.364, 1.03)	0.062
≥ 28	0.504 (0.31, 0.505)	0.006
Infertility duration (years) (< 1 vs ≥ 1)	1.02 (0.984, 1.058)	0.284
Infertility type (primary vs secondary)	1.039 (0.83, 1.3)	0.74
Infertility cause		
Tubal	reference	
Decreased ovarian deserve	0.791 (0.593, 1.057)	0.113
Endometriosis	0.624 (0.304, 1.279)	0.198
Male factors	0.956 (0.678, 1.348)	0.799
Others	1.120 (0.762, 1.646)	0.564
Endometrium thickness (mm)	1.054 (1.001, 1.109) per every 1-mm increase	0.045
Embryo transfer number (2 vs 1)	2.454 (1.846, 3.261)	0
Embryo stage (blastocyst vs cleavage)	1.362 (0.997, 1.898)	0.082
Embryo quality (good vs poor)	1.035(1.012, 1.081)	0.038
cryopreservation time	0.999 (0.997, 1.000) per every 1-year increase	0.126
LH surge (yes vs no)	0.947 (0.769, 1.166)	0.607

## Discussion

The results from the current study showed similar pregnancy outcomes, including implantation rate, clinical pregnancy rate and live birth rate, in women having a spontaneous LH surge vs. not in mNC-FET. Notably, FET was conducted 1 day earlier in the LH surge group than in the non-LH surge group. For cleavage embryos, FET was conducted 4 and 5 days after hCG injection in women with and without LH surge, respectively. For blastocysts, FET was conducted 6 and 7 days after hCG injection in women with and without LH surge, respectively. Multivariate regression analysis showed that live birth was not associated with spontaneous LH surge.

Consistent with previous studies, the LH surge was characterized as a higher serum LH level, accompanied by a lower estradiol and higher progesterone level. However, the cut-off of LH level remained debated in the literature, which was an important parameter to predict the time of ovulation [26]. The cuto-ff level (≥ 20 IU/L) used in the current study was also used in many previous studies [12, 27] and based on previous internal clinic outcomes [6, 28]. The associated endocrine events in the LH surge group compared with without LH surge was in line with the LH surge definition in our study. In line with this rationale, the rate of spontaneous LH surge occurred close to 50% before hCG trigger, which was similar to a prospective trial finding [29], but the LH surges ≥ 10 IU/L in that trial. Bartels et al. found no difference in the ongoing pregnancy rate with varying threshold from 15 to 25 IU/L for LH surge [27]. Irani et al. defined LH surge based on the first attainment of LH ≥ 17 IU/L combined with a ≥ 30% drop in estradiol on the following day, and achieved better NC-FET pregnancy outcomes [30]. However, all these studies were retrospective and different in the study design. Therefore, the optimal LH cut-off value remained controversial.

Using the ≥ 20 IU/L definition for LH surge, our findings are consistent with the result of a previous retrospective study [31, 32], pregnancy outcomes were not significantly different in patients with LH surge vs. without LH surge on the day of hCG trigger. However, these studies had a small sample size. A study by Johal et al. observed similar pregnancy outcomes in the mNC-FET cycle if FET was conducted on LH/HCG + 6 in cycles with LH surge (≥ 20 IU/L) but on HCG + 7 in cycles without LH surge [32]. Although the FET timing and LH surge cut-off in that study were the same as those in our study, the timing of exogenous progesterone administration was 1 day later compared with our study, which might be associated with the luteal support after FET. In a study by Kahraman et al., pregnancy outcomes did not differ between cycles with different LH levels on the hCG day, but the low LH level was measured the day before hCG rather than the hCG day [31]. Such a difference might have an impact on pregnancy outcome due to sub-optimal endometrial receptivity.

Majority of previous studies that compared the spontaneous LH surge and hCG induced ovulation in the natural FET cycle obtained comparable pregnancy outcomes [12–16, 33]. In these studies, hCG was not used if spontaneous LH surge occurred. In the current study, however, hCG was used regardless of the presence of spontaneous LH surge or not. Such a choice was made based on several reasons. First and foremost, LH surge in natural cyclew is highly irregular. Direito et al. found significant variation in the amplitude, duration, and mode of LH peak [34]. LH peak may be single, double, multiple, or even absent [35, 36]. Cahill et al. showed that LH peak starts from midnight to 8 am in majority of the women, but varies significantly among individuals, and even among different cycles of the same individuals [37]. Duration of LH peak also varies significantly. In the study by Direito et al., the average duration of LH peak was 4.8 days [34]. In other words, timing of ovulation relative to the spontaneous LH surge is uncertain. An artificial cycle for the endometrial preparation is also preferred in most reproductive clinics for this reason. HCG application could simulate the LH surge more accurately and better synchronize the embryo development with endometrium. Second, hCG may provide support to corpus luteum development and stimulate endometrium via the VEGF pathway, ultimately improving pregnancy outcomes [38]. Bienert et al. found hCG could increase the expression of endothelial cell–cell adhesion molecules to improve the implantation and pregnancy rate [39]. Finally, hCG administration could reduce patient visits to the hospital for ultrasound scans and frequent blood hormone to exam LH surge [14].

In the multivariate regression analysis in the current study, live birth was associated positively with transfer of two embryo (versus only one) and good embryo quality, and negatively with older maternal age, higher BMI, and lower endometrium thickness. These findings were consistent with previous studies [4, 28, 40]. Also consistent with previous studies, live birth was not associated with LH surge.

A strength of this study is the relatively large sample size. This allows us to use multivariate regression to control confounding factors analysis. The second strength lies in the consistent approach to synchronize embryo and endometrium between the two groups. Briefly, exogenous progesterone supplementation started 1 day after the theoretical ovulation day. Embryo transfer was conducted 2 days later for cleavage embryos and 4 days later for blastocysts after exogenous progesterone supplementation. As a result, the timing relative to the theoretical ovulation day is identical, and the duration of progesterone exposure from exogenous progesterone supplementation to the embryo transfer is comparable in two groups.

As a retrospective analysis, a key limitation in the current study is missing information. Second, all cases were from a single centre. Whether the results are applicable to the general setting remains unknown. Third, we did not examine ovulation with ultrasound after hCG administration, which in turn might affect pregnancy outcomes, as suggested by the lower live birth rate with artificial cycle than natural cycle and stimulation cycle because of lack of corpus luteum [6]. We did, however, examine blood progesterone level on the FET day, and the results suggested sufficient luteal support after exogenous progesterone supplementation. Fourth, endogenous progesterone was not measured on the endometrium transformation day and cycles. Cycles with progesterone > 1.0 ng/ml on the hCG day were excluded, since elevated progesterone suggested an asynchrony between endometrium and embryo development. However, a more recent study by Bartels et al. failed to show any effects of increased progesterone (≥ 1 ng/ml at 6 days before transfer) on pregnancy outcomes [27]. Lee et al. also found that progesterone > 5 nmol/L on the LH surge day did not have an adverse effect on pregnancy outcome [41]. Prospective cohort studies are needed to further examine the potential impact of spontaneous LH surge on live birth in mNC-FET.

## Conclusion

In conclusion, pregnancy outcomes with FET cycles did not differ in subjects with vs. without spontaneous LH surge in modified natural cycle if FET timing was adjusted. This finding suggest that there is no need to wait for a LH surge if the leading follicle has matured.

## Data Availability

The datasets used and/or analyzed during the current study are available from the corresponding author on request.

## Abbreviations

IVF: In vitro fertilisation
ICSI: Intracytoplasmic sperm injection
COS: Controlled ovarian stimulation
hCG: Human chorionic gonadotropin
OS: Ovulation stimulation
mNC: Modified natural cycle
FET: Frozen embryo transfer
FSH: Follicle-stimulating hormone
LH: Luteinising hormone
MC: Menstrual cycle
SD: Standard deviation
aORs: Adjusted odds ratios
CIs: Confidence intervals
BMI: Body mass index
